# The RND1 Small GTPase: Main Functions and Emerging Role in Oncogenesis

**DOI:** 10.3390/ijms20153612

**Published:** 2019-07-24

**Authors:** Laetitia Mouly, Julia Gilhodes, Anthony Lemarié, Elizabeth Cohen-Jonathan Moyal, Christine Toulas, Gilles Favre, Olivier Sordet, Sylvie Monferran

**Affiliations:** 1 Cancer Research Center of Toulouse, INSERM UMR1037, 31037 Toulouse, France; 2 Faculty of Pharmacy and Medecine, Université Toulouse III, 31062 Toulouse, France; 3 Institut Claudius Regaud, IUCT-O, 31059 Toulouse, France

**Keywords:** Rho GTPase, RND, migration, oncogenesis, response to anticancer agents, prognosis factor

## Abstract

The Rho GTPase family can be classified into classic and atypical members. Classic members cycle between an inactive Guanosine DiPhosphate -bound state and an active Guanosine TriPhosphate-bound state. Atypical Rho GTPases, such as RND1, are predominantly in an active GTP-bound conformation. The role of classic members in oncogenesis has been the subject of numerous studies, while that of atypical members has been less explored. Besides the roles of RND1 in healthy tissues, recent data suggest that RND1 is involved in oncogenesis and response to cancer therapeutics. Here, we present the current knowledge on RND1 expression, subcellular localization, and functions in healthy tissues. Then, we review data showing that RND1 expression is dysregulated in tumors, the molecular mechanisms involved in this deregulation, and the role of RND1 in oncogenesis. For several aggressive tumors, RND1 presents the features of a tumor suppressor gene. In these tumors, low expression of *RND1* is associated with a bad prognosis for the patients. Finally, we highlight that *RND1* expression is induced by anticancer agents and modulates their response. Of note, *RND1* mRNA levels in tumors could be used as a predictive marker of both patient prognosis and response to anticancer agents.

## 1. Introduction

### RND1, an Atypical Rho GTPase

**Figure 1:**
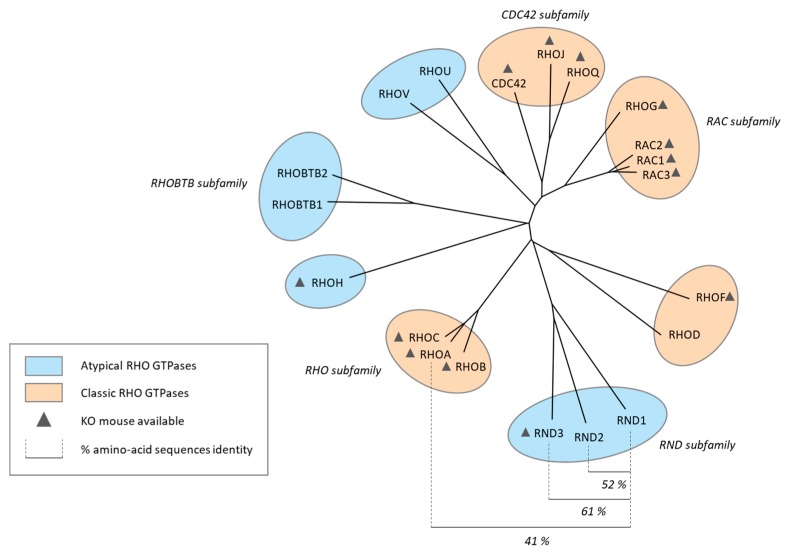
Rho GTPase family. The phylogenetic tree of the Rho GTPase family was obtained from the alignment of the amino-acid sequences of the 20 Rho GTPases using the Clustal Omega program. The tree was generated using the iTOL software. Rho members are structured into eight subfamilies, including the Rho subfamily (RHOA, RHOB, and RHOC), the RAC subfamily (RAC1, RAC2, RAC3, and RHOG), the CDC42 subfamily (CDC42, RHOQ and RHOJ), the RND subfamily (RND1, RND2 and RND3), the RHOU/V family (RHOU and RHOV), the RHOD/F subfamily (RHOD and RHOF), the RHOBTB subfamily (RHOBTB1 and RHOBTB2) and RHOH. Classic Rho GTPases comprise the RHO, RAC, CDC42, and RHOD/F subfamilies. Atypical Rho GTPases include the RND, RHOU/V, RHOBTB, and RHOH subfamilies. Rho GTPases, which have a knockout (KO) mouse model, are annotated with a triangle. Percentage of amino-acid-sequences identity between RND1 and other Rho GTPases members were determined with the Clustal Omega tool.

**Figure 2:**
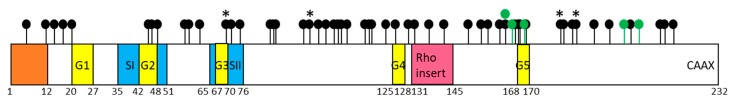
Structure of *RND1* gene and mutational pattern in human tumors. The orange box is the lipid raft motif; the yellow boxes are the guanosine nucleotide binding sites; the pink box is Rho insert domain; the blue boxes are the switch domains. SI: Switch I; SII: Switch II. Mutation types and corresponding color codes are as follows: Black represents the missense mutations and green represents the nonsense mutations. The stars indicate the mutations for which the impact on the RND1 function has been studied [10].

The Rho family of GTPases includes 20 members, which can be classified into classic and atypical members in humans [1,2] (Figure 1). Classic Rho GTPases, such as RHOA, CDC42, and RAC1, act as molecular switches by cycling between an inactive Guanosine DiPhosphate (GDP)-bound state and an active Guanosine TriPhosphate (GTP)-bound state. The GDP/GTP cycle depends on guanine nucleotide exchange factors (GEFs) and GTPase-activating proteins (GAPs). GEFs facilitate the exchange of GDP with GTP while GAPs promote hydrolysis of GTP into GDP by stimulating the intrinsic GTPase activity of Rho. Atypical Rho GTPases are primarily in an active GTP-bound conformation by two main mechanisms [3,4]. RHOU, and probably also RHOV, have a high nucleotide exchange rate and are therefore considered to be mainly GTP-bound [4]. Due to some substitutions in residues critical for GTP hydrolysis, which correspond to Gly14, Ala61, and Gln63 of RHOA, RND proteins are unable to hydrolyze GTP and consequently, are always activated [3]. RND1 (RHO6) belongs to the RND subfamily that also comprises RND2 (RHO7/RHON) and RND3 (RHO8/RHOE) (Figure 1). Human RND members share 52–61% homology in their primary sequences (Figure 1). RND proteins possess the Rho-specific insert domain (Figure 2), which defines the family of Rho GTPases. Although RND1 shares 41% identity with RHOA protein (Figure 1), RND proteins differ from other Rho GTPases by several features. They contain an extension of 30 amino acids at the C-terminal and RND1 and RND3 proteins possess 8 and 18 additional amino acids, respectively, at the N-terminal [3]. At the N-terminal, RND1 and RND3 (but not RND2) contain a KERRA (Lys-Glu-Arg-Arg-Ala) sequence that mediates their targeting to lipid rafts, and so their localization at the plasma membrane [5]. Concerning post-translational prenylation, unlike the proteins of the RHOA, RAC1, and CDC42 subfamilies (see Figure 1), which are predominantly geranyl-geranylated, RND proteins are farnesylated due to a methionine in the C-terminal CAAX box. The activity of RND proteins is rather dependent on epigenetic, transcriptional, post-transcriptional, and post-translational mechanisms, than on GDP/GTP exchange [6]. Finally, when bound to GTP, both classic and atypical Rho GTPases bind to their effectors and activate signaling pathways that regulate various cellular processes, such as cytoskeleton dynamic, proliferation, and cell migration [7,8].

Dysregulation of classic Rho GTPases activity can lead to dysregulation of processes important for tumorigenesis, such as metastasis or angiogenesis capacity [9]. Data from the literature suggest that RND1 [10] and RND3 [11,12] play an essential role in oncogenesis and in response to therapeutics. Insofar as these data have been recently well reviewed for RND3 [13], we will focus our discussion on RND1. First, we will review current knowledge on the expression, localization, regulation, and functions of RND1 in healthy tissues. Then, we will review its expression in cancers, its role in oncogenesis, and its involvement in the response to anticancer agents.

## 2. RND1 Expression in Healthy Tissues

Unlike RND3 whose expression is ubiquitous in humans, RND1 is not expressed in all tissues. During embryogenesis, *RND1* mRNA is expressed in various human fetal tissues, such as the brain, lungs, liver, and kidneys [3]. In Xenopus, it has been shown that the expression of RND1 varies during the different stages of embryogenesis. *RND1* mRNA is particularly expressed during the gastrulation in the ectoderm and the dorsal lip of the blastopore. During neurulation, *RND1* mRNA is expressed in the somitogenic mesoderm. At mid-neurula, *RND1* transcript is expressed in the somites and in cells forming the neural crest, then decreases after their formation is completed [14].

In adults, human *RND1* mRNA is expressed in the liver and several brain tissues and more weakly, in the lungs, pancreas, thymus, prostate, ovaries, and small intestine [3]. In rats, RND1 protein is expressed in the brain, liver, and testis, but not in the muscle [3]. In the rat brain, RND1 protein is strongly expressed in cortical and pyramidal neurons of the hippocampus during synaptic formation [15]. At present the origin of the variation of RND1 expression in tissues is not known. It is conceivable that RND1 may act at very low levels or may control cellular processes specific to particular tissues, such as the brain. It will be described below in the section “Biological Functions of RND1”.

During pregnancy, human and rat *RND1* mRNA increases in myometrium within the uterus [16,17]. In rats, the expression of *RND1* mRNA increases until it reaches a maximum value at the end of gestation (21 days). This *RND1* mRNA induction depends at least on estradiol and progesterone levels [16]. From the first postpartum day, the expression of *RND1* transcript in myometrium immediately falls back to the initial level.

## 3. Subcellular Localization of RND1

### Subcellular Localization

**Figure 3:**
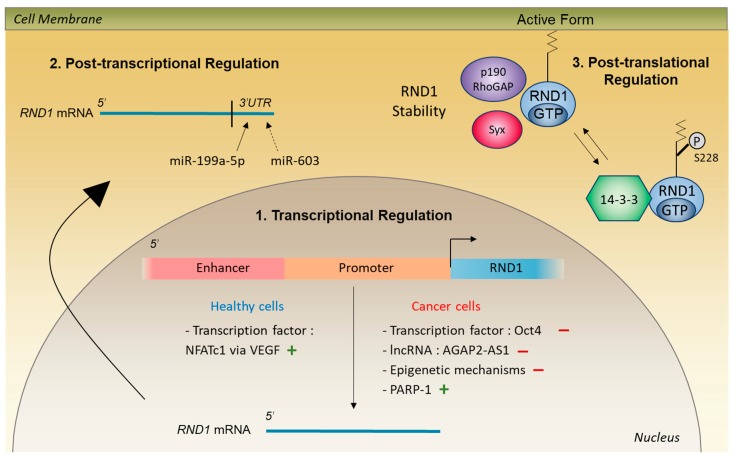
Regulation of RND1 protein in healthy and cancer cells. 1: *RND1* transcription is regulated by diverse mechanisms. In healthy cells, the transcription factor NFATc1 increases *RND1* transcription (marked as “+” in the figure). In cancer cells, the transcription factor Oct4, the lncRNA AGAP2-AS1 and epigenetic mechanisms, including DNA methylation and histone acetylation, act as repressors of *RND1* transcription (marked as “−” in the figure) whereas PARP-1 stimulates *RND1* transcription (marked as “+” in the figure). 2: *RND1* expression is inhibited by the miRNAs miR-199a-5p and miR-603. The 3′UTR of RND1 mRNA has one or more specific binding sites for miR-199a-5p whereas the binding sites for miR-603 have not yet been identified. 3: RND1 protein is stabilized when it associates with its effectors p190RhoGAP and Syx. When RND1 is phosphorylated, it binds to the 14-3-3 protein, which induces the translocation of RND1 from the plasma membrane to the cytoplasm.

In rats, the endogenous RND1 protein has been detected in the dendrites and the axon of hippocampal neurons [15]. To date, no commercial antibodies have been reported to detect the endogenous human RND1 protein. Thus, to study the subcellular protein localization, transfections of cells with plasmid containing the coding sequence of *RND1* were performed. In these models of RND1 overexpression, active RND1 is localized at the plasma membrane [3,14,18] due to its N-terminal extension [5] and its farnesylation [19]. Indeed, deletion of the first seven amino acids of RND1 or treatment with an inhibitor of farnesyl transferase results in a decrease of RND1 at the plasma membrane and its accumulation in the cytoplasm and the nucleus [5,19]. RND1 may be also sequestrated in the cytosol, in a phosphorylated form, when it is bound to 14-3-3 protein [20], which can be considered as an inhibitor of RND1 activity. The kinase that phosphorylates RND1 is unknown (Figure 3).

## 4. Control of *RND1* Expression in Healthy Cells

Depending on the growth factors, the expression of *RND1* can be induced or repressed. *RND1* mRNA is decreased by osteoprotegerin in osteoclasts [21] and by various mitogens, such as EGF, insulin, hydrocortisone, or bovine pituitary extracts in mammary MCF-10A epithelial cells [10]. On the contrary, *RND1* transcript expression is induced by different growth factors, such as TGF-β (transforming growth factor beta) or VEGF (vascular endothelial growth factor) at short times (between 1 h and 2 h) in MCF10A or endothelial cells [10,22]. The transcriptional increase of *RND1* by VEGF is due to the binding of the NFATc1 (Nuclear Factor of Activated T Cells 1) transcription factor to *RND1* promoter and to a distal enhancer region of *RND1* gene [22]. Moreover, the expression of *RND1* mRNA is also induced by hormones, such as estradiol and progesterone [16,23].

Because RND1 is always in its active GTP-bound form, it is mainly regulated by modifications of its expression. Below are detailed the levels of regulation that have been reported, including transcriptional, post-transcriptional, and post-translational mechanisms, which are summarized in Figure 3.

### 4.1. Transcriptional and Post-Transcriptional Regulation

Concerning the regulation by transcription factors, the binding of the NFATc1 protein to the promoter and to a distal enhancer region of *RND1* gene, in response to VEGF, increases the transcription of *RND1* in endothelial cells [22]. Regarding the post-transcriptional regulation, overexpression of miR-199a-5p in immortalized urothelial TEU-2 cells results in a decrease of *RND1* mRNA [24]. The 3′UTR of *RND1* mRNA possesses at least one specific binding site for miR-199a-5p. In addition, overexpression of miR-603 in human cervical cancer HeLa cells and in human embryonic kidney HEK293 cells decreases *RND1* mRNA [25].

### 4.2. Post-Translational Regulation

Several Rho GTPases are regulated by phosphorylation [26]. When phosphorylation occurs near the CAAX box, it alters the localization and function of Rho GTPases [26]. The binding of the phosphorylated form of RND1 on S228, close to the CAAX box, to 14-3-3 protein induces the translocation of RND1 from the plasma membrane to the cytoplasm and reverses the typical RND1 rounded phenotype described below in the section “Biological Functions of RND1” [20]. The action of 14-3-3 on RND1 is similar to the action of GDIs (GDP-dissociation inhibitors) on classic GTPases. GDIs relocate CDC42 and RHOA from their site of action at the plasma membrane to the cytoplasm in order to prevent interaction with their effectors. The kinase that phosphorylates RND1 is not known. In addition to these phosphorylation processes, the interaction of RND1 with its effectors p190RhoGAP and Syx, a RHOA GEF, increases RND1 stability [27].

## 5. Biological Functions of RND1

### 5.1. Regulation of Cell Morphology and Actin Cytoskeleton

The first cellular effect reported for RND1 (and RND3) is on cell morphology by reducing actomyosin contractibility [3]. These observations are at the origin of the name of the subfamily RND. RND comes from “round” because the overexpression of RND1 or RND3 in fibroblasts results in a rounded cell phenotype [3]. The expression of RND1 induces the loss of stress fibers and focal adhesions helping to anchor the cells to the extracellular matrix. In consequence, cells tend to contract. However, they maintain some contact with the matrix, which gives them a dendritic phenotype [3].

The first mechanism described to explain how RND1 reduces actomyosin contractibility is through RHOA inhibition [28]. RND1 (and RND3) can bind the central domain of p190RhoGAP, one of RHOA’s most abundant GAPs. This interaction increases the GAP activity of p190 towards RHOA resulting in a decrease of RHOA active form and thus, an inhibition of stress fiber formation [28]. As a regulatory mechanism, the RHOA effector ROCK1 can phosphorylate p190RhoGAP, which decreases p190RhoGAP binding to RND1, so preventing the inhibitory effect of RND1 on RHOA [29].

The regulation of actin cytoskeleton by RND1 could also involve RND1 partners. RND1 can bind Syx [30], which activates RHOA. In the same way as for RND3 [30], binding of RND1 to Syx may cause Syx inhibition and in turn RHOA inhibition. A RND1 partner, Socius, was identified by the yeast two-hybrid technique using RND1 as bait [18]. The expression of Socius in a form allowing it to be addressed to the plasma membrane (Socius-CAAX) causes a loss of stress fibers [18]. Moreover, in cells co-transfected with Socius-CAAX and RND1, the expression of Socius reverses the typical RND1-dependent rounded phenotype. RND1 also interacts with the SH2 domain of Grb7 (Growth factor receptor-bound protein 7), a protein that belongs to the family of adapter proteins involved in signal transduction [31] and that stabilizes actin filaments [32]. At present, the biological effects of this interaction have not been explored. We could consider that by interacting with Grb7, RND1 may inhibit it and prevent it from activating actin stress filaments.

### 5.2. Formation of Nerve Connections

Cell polarity is essential for neuronal development and is related to changes in the actin cytoskeleton [33]. The growth cone is a structure rich in filamentous actin (actin F), located at the end of the growing axon, which connects the axon to the target neuron. A network of microtubules allows the expansion of the growth cone. The neurites—filopodia and lamellipodia—that emanate from the growth cone explore the environment in all directions. The growth cones express receptors for guide molecules and, depending on the signals received, alternate between elongation and retraction of the filopodia and lamellipodia. Rho proteins, including RND1, participate in the formation of nerve connections.

#### 5.2.1. Interaction with Plexins

**Figure 4:**
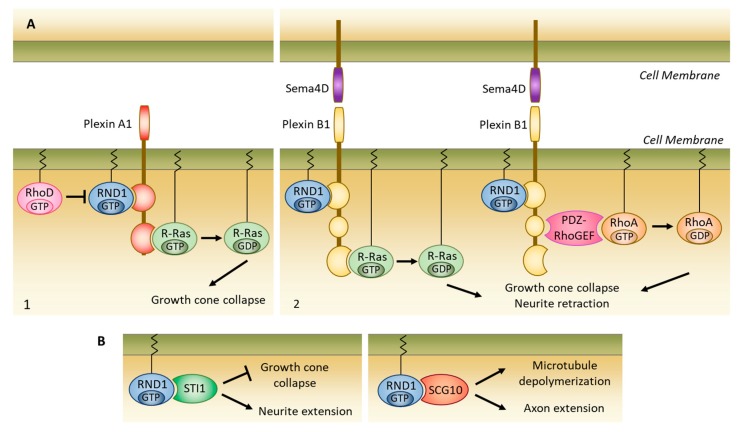
RND1 is involved in the formation of nerve connections. (**A**) Plexin dependent mechanisms. 1: The interaction of RND1 with plexin A1 stimulates its R-Ras GTPase activity, leading to the growth cone collapse. This effect occurs even in the absence of a semaphorin signal. This interaction can be inhibited by RhoD, which binds the plexin to the same binding site as RND1. 2: After stimulation by semaphorin 4D (Sema4D), the interaction of RND1 with plexin B1 stimulates its R-Ras GTPase activity and its binding with PDZ-RhoGEF, resulting in the inhibition of R-Ras and the activation of RhoA, respectively. These mechanisms both contribute to growth cone collapse and neurite retraction. (**B**) Plexin independent mechanisms. The interaction of RND1 with STI1 inhibits growth cone collapse inhibition induced by the RND1-Plexin A1 association and increases neurite extension. Binding of RND1 with SCG10 induces microtubule depolymerization and axon extension.

RND1 can interact with different types of plexins, which are receptors for the guide molecules semaphorins. Binding of semaphorin 3A (Sema3A) ligand to neuropilin 1 (NRP1)/plexin A1 complex induces growth cone collapse. The interaction of RND1 with plexin A1 also induces the growth cone collapse even in the absence of Sema3A or NRP1 co-receptor [34] (Figure 4A). The effect of RND1 is related to the stimulation of the GTPase activity of plexin A1, which is responsible for a decrease in R-Ras activity [35]. The interaction of RND1 with plexin A1 can be inhibited by RHOD, which binds to the same binding site as RND1 [34].

In Cos-7 cells, co-expression of RND1 and plexin B1 causes cell contraction after stimulation of plexin B1 by its ligand, semaphorin 4D (Sema4D). This effect depends on two independent mechanisms (Figure 4A). The first is linked to the increase of RHOA activity. Indeed, the binding of RND1 to plexin B1 induces the interaction of PDZ-RhoGEF to plexin B1 and its PDZ-RhoGEF activation. Consequently, PDZ-RhoGEF activation increases the activation of RHOA [36]. Similarly to plexin A1, the second mechanism depends on the inhibition of R-Ras activity. RND1 induces the binding of plexin B1 with R-Ras in its GTP-linked active form. After stimulation with Sema4D, the GAP domain of plexin B1 stimulates the GTPase activity of R-Ras and, therefore, inhibits R-Ras activation [35,37]. In Cos-7 cells, co-expression of RND1 and plexins B2 or B3 also induces cell contraction after Sema4D stimulation [38].

#### 5.2.2. Interaction with Other Partners

RND1 also regulates neuronal polarity by plexin-independent mechanisms. (i) In pheochromocytoma PC-12 cells, depletion of RND1 with siRNA suppresses fibroblast growth factor (FGF)-induced neurite extension [39]. The effect of RND1 on neurite extension is linked both to its binding to the FRS2β protein (FGFR substrate 2 β) and to its inhibitory function on RHOA [40]. (ii) RND1 can interact with STI1 (Stress-Inducible Protein), a co-chaperone protein that associates with HSP70/HSP90 [41]. This interaction inhibits the growth cone collapse induced by RND1-Plexin A1 interaction in Cos-7 cells and increases neurite extension in PC-12 cells (Figure 4B). (iii) RND1 binds SCG10 (Superior Cervical Ganglion 10), a protein that destabilizes neuronal microtubules in the growth cone and that is essential for axon extension (Figure 4B) [42]. This binding enhances the effects of SCG10 on microtubule depolymerization and induces axon extension in rat hippocampal neurons.

Depending on the type of effector that RND1 connects, RND1 activates or decreases neurite extension and induces or inhibits the growth cone collapse.

#### 5.2.3. Role in Embryonic Development

**Figure 5:**
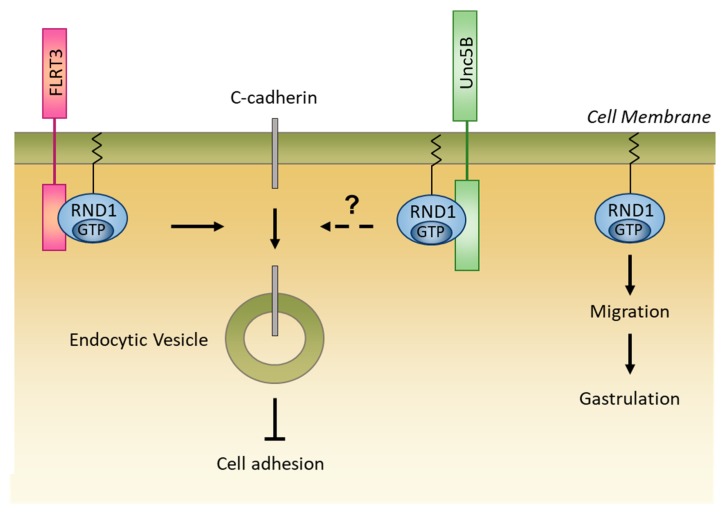
RND1 is involved in adhesion and migration during embryogenesis. In Xenopus embryos, RND1 can bind both the FLRT3 transmembrane protein and the netrin receptor, Unc5B. The association between RND1 and FLRT3 decreases the level of C-cadherin present on the cell surface through internalization of C-cadherin into endocytic vesicles in the cytoplasm, resulting in cell adhesion inhibition. The effect of the interaction with Unc5B on the internalization of C-cadherin has not yet been studied. RND1 also allows endo-mesodermal cells to migrate during embryogenesis, which leads to gastrulation.

Depletion of RND1 with morpholino antisense oligonucleotide (MO) in four-cell stage Xenopus embryos (into the equatorial zone) induces aberrant gastrulation movements, i.e., the leading endo-mesordermal cells fail to crawl under the ectoderm [43] (Figure 5). As a consequence, at high concentrations of RND1 MO, embryos fail to gastrulate and sometimes die. At lower concentrations of RND1 MO, the migration of endo-mesordermal cells is delayed, resulting in bent embryos. In addition to the intrinsic effect of RND1 on morphogenesis, RND1 participates in morphogenesis via its interaction with FLRT3 transmembrane protein (Fibronectin Leucine Rich Transmembrane 3) and with the netrin receptor, Unc5B [43,44]. During development, the interaction between RND1 and FLRT3 inhibits cell adhesion [14,43] by decreasing the level of C-cadherin present at the plasma membrane through internalization of C-cadherin in the cytoplasm [43] (Figure 5). The effect of the interaction with Unc5B on the internalization of C-cadherin has not yet been explored.

#### 5.2.4. Role in Angiogenesis

**Figure 6:**
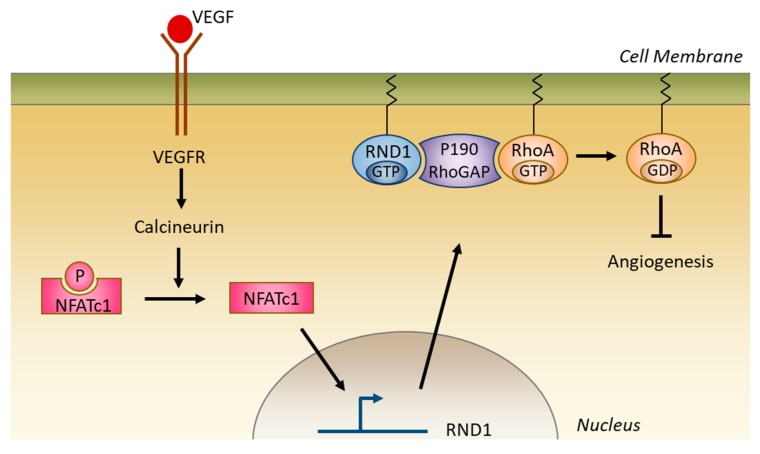
RND1 limits angiogenesis. Vascular endothelial growth factor (VEGF) activates calcineurin-NFATc1 signaling axis. Nuclear localized NFATc1 binds to the *RND1* promoter and stimulates its transcription. The increase of RND1 decreases RHOA activation resulting in the inhibition of angiogenesis.

RND1 appears to limit angiogenesis. Depletion of RND1 with siRNA in endothelial cells leads to an increase in VEGF-mediated endothelial migration and in the formation of neo-vessels (Figure 6) [22]. It has been proposed that in endothelial cells, VEGF induces the expression of *RND1*, which restrains the activity of RHOA and thus limits RHOA positive effects on angiogenesis [22].

## 6. RND1 Expression in Cancers

### RND1 Expression is Altered in Cancers

As other Rho GTPases [2], the expression of *RND1* transcripts is altered in cancers. *RND1* is down-regulated in the most aggressive subtypes of breast cancers (estrogen receptor negative, triple negative, and basal) [10], in hepatocellular carcinoma [45,46] and in high grade glioma [11,47]. Its expression is also lower in the advanced stages (grade III/IV) than in the early stages (grade I/II) of hepatocellular carcinoma [45] and glioma [11]. By contrast, *RND1* transcripts are up-regulated in low grades of breast tumors [48], in gastric cancer cell lines [49], and in esophageal squamous cell carcinoma [50].

The low expression of *RND1* in tumors was first described as being due to genetic and epigenetic mechanisms. The 12q12-q13 region where *RND1* gene is located is deleted in several cancers, such as pancreas cancer [51], adenoid cystic carcinoma [52], and breast cancer [10]. In adenoid cystic carcinoma [52] and in breast cancer [10], the deletion of *RND1* gene concerns only one of the two alleles. In several tumors, it has been reported that methylation of *RND1* promoter and histone deacetylation induce the repression of *RND1* gene transcription [10,46,53,54] (Figure 3). Moreover, in breast cancers, the Polycomb Repressor Complex 2 is involved in the inhibition of *RND1* gene expression [10]. Another mechanism responsible for the low expression of *RND1* in tumors is the inhibition of its transcription through the deregulation of transcription factors (Figure 3). In human breast cancers, the Oct4 transcription factor is increased compared to normal breast tissues [55] and is over-expressed in metastatic breast tumors compared to primary breast tumors [56]. Oct4 binds to the promoter of *RND1* on two sites (−844 and −1271 bp) and this binding inhibits *RND1* transcription in the human cancer MDA-MB231 cell line [57]. More recently, long non-coding RNA (lncRNA) have been involved in the inhibition of *RND1* expression (Figure 3). The lncRNA AGAP2-AS1 has been identified as a repressor of *RND1* [58,59]. Inhibition of AGAP2-AS1 expression with siRNA increases *RND1* mRNA in non-small lung cancer H1975 cells and gastric cancer BGC823 cells. High expression of AGAP2-AS1 in non-small cell lung cancers and gastric cancers is associated with tumor progression. These data raise the possibility that *RND1* may have an anti-tumor function in these cancers.

Regarding the induction of *RND1* expression in cancers, there are fewer data in the literature. Due to hypomethylation of *RND1* gene, *RND1* is overexpressed in gastric cell lines [49]. However, the consequences of this increase on oncogenesis are not known yet.

Analysis of the genomic database in the cbioportal website (http://www.cbioportal.org) reveals that several mutations in the *RND1* open reading frame exist in human tumors (Figure 2 and Table 1). The majority of mutations are missense mutations (124 mutations). Sixteen truncating mutations and two fusion proteins have been discovered (Table 1). Unlike *RAC1* and *RHOA* gene mutations, which occur preferentially in the G1 guanosine nucleotide binding site and the switch I and II domains [2], *RND1* mutations spread over almost the entire gene sequence (Figure 2). The impact on RND1 function of four missense mutations discovered in breast tumors has been studied. Unlike wild type RND1, G70R, E98D, and F180C mutants are not able to induce a growth inhibitory activity, show an aberrant localization in the cytoplasm, and lose their ability to bind to plexin B1 [10]. The M185V RND1 mutant behaves similarly to the wild type in terms of subcellular localization and interaction with plexin B1. The impact of other *RND1* mutations on its function and tumor initiation and progression remains to be determined.

## 7. RND1 in Oncogenesis

The alterations of *RND1* expression in tumors and an in silico analysis that identified RND1 as a tumor suppressor gene [60] suggest a potential role of RND1 in oncogenesis. In breast tissue, the loss of RND1 induces tumorigenesis in mammary epithelial cells [10]. On the contrary, the re-introduction of *RND1* gene in mammary tumor cells slows down the tumor growth [10]. The loss of *RND1* in mammary epithelial cells also contributes to tumor progression via (i) the induction of epithelial to mesenchymal transition (EMT), (ii) the induction of proliferation, and (iii) the ability to migrate and metastasize. These tumor progression effects induced by *RND1* depletion are mediated through the activation of the Ras and MAPK pathways [10].

The role of *RND1* in EMT has been extended to hepatocellular carcinoma cells, in which the depletion of *RND1* also causes EMT, this time via the activation of RHOA [46]. In glioblastoma initiating cells, the expression of *RND1* is inversely correlated with mesenchymal gene expression, such as TWIST1, SNAI1, and SNAI2 [47], suggesting that *RND1* blocks the mesenchymal phenotype.

Concerning the role of *RND1* in tumor cell proliferation and migration, the results seem to be dependent on the tumor type. The depletion of *RND1* induces hepatocellular carcinoma proliferation [45], whereas it decreases esophageal squamous cell carcinoma proliferation [50]. The silencing of *RND1* increases the in vitro migration and invasion of glioblastoma initiating cells [47] and hepatocellular carcinoma cells [45,46]. In hepatocellular carcinoma cells, the increase of invasion induced by *RND1* depletion is mediated by the activation of RHOA [46]. In contrast to these data, the silencing of *RND1* in esophageal carcinoma cells decreases their in vitro migration and metastasis capacity [50]. All these proliferation and migration data show that a dysregulation of the amount of RND1 (either overexpression or decreased expression) in cancer cells increases the proliferation and migratory capacity of these cells. In fact, as described in the previous section, *RND1* is down-regulated in hepatocellular carcinoma, in the most aggressive subtypes of breast cancers and in glioblastoma patients, whereas it is over-expressed in esophageal squamous cell carcinoma, which could explain the contradictive effects of its variation on cell behavior.

## 8. RND1 In Vital Prognosis

*RND1* has been described as a prognosis factor of survival for patients with an estrogen receptor negative breast cancer [10], with hepatocellular carcinoma [45,46] or glioblastoma [47]. In these tumors, a weak expression of *RND1* is correlated with a bad prognosis. In glioblastoma, we identified an *RND1*^low^ signature of six genes (ITGA5, COL3A1, COL5A1, MET, COL1A2, LAMC1), upregulated in glioblastoma patients with low *RND1*, that predicts the overall survival of glioblastoma patients. Moreover, by multivariate statistical analysis that tests its independence from other known prognostic factors, we determined that in glioblastoma the *RND1*^low^ signature is an independent prognostic factor [47]. This *RND1*^low^ signature may be helpful in clinical practice to predict the survival of glioblastoma patients. This signature might also lead to clinical application to optimize the treatment of glioblastoma through the targeting of genes involved in this signature.

## 9. *RND1* in Response to Anticancer Agents

### RND1 Expression and Its Role in Response to Anticancer Agents

Screening approaches on chips have shown that *RND1* expression is increased in response to anticancer agents. Co-treatment of chronic myeloid leukemia cells with imatinib, a tyrosine kinase inhibitor of Bcr-Abl protein, and the antioxidant agent amifostine, increases *RND1* mRNA levels after 48 h [61]. *RND1* mRNA is also induced in human lymphocytes 15 min after ionizing irradiation [62]. Using a RT-qPCR screen of Rho GTPase expression in response to the topoisomerase I inhibitor camptothecin, we identified *RND1* as a Rho GTPase gene, which is rapidly induced in the osteosarcoma U2OS cell line [53]. The poly (ADP-ribose) polymerase (PARP-1) activity is responsible for the induction of *RND1* transcription after treatment with camptothecin (Figure 3). This rapid induction of *RND1* is also found in several non-tumorigenic cells, such as NIH3T3 fibroblasts, and in other tumor cells, such as U87 glioblastoma cells. In contrast, the expression of *RND2* and *RND3*, the two *RND1* homologs, is not induced after a short treatment with camptothecin [53].

There are currently few data on the effect of *RND1* expression on cell sensitivity to anticancer agents. In hepatocellular carcinoma, *RND1* depletion with siRNA induces the resistance of hepatocellular carcinoma cells to cisplatin, a water-soluble platinum complex [45], whereas *RND1* re-expression induced by cell treatment with an inducer of promoter hypomethylation and an histone deacetylase inhibitor increases the cellular sensitivity to the Raf inhibitor, sorafenib [46]. The induction of *RND1* protects osteosarcoma cells against camptothecin, likely by inhibiting apoptosis [53]. From these data, we can conclude that the effect of RND1 on cell sensitivity appears to depend on the cell type or the drug used.

**Figure 7:**
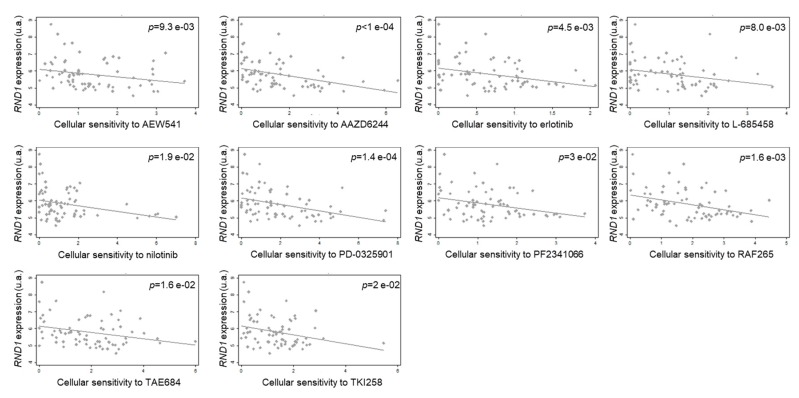
Correlation between *RND1* expression and sensitivity to anticancer agents in hematological and lymphoid tumor cells. Using the CCLE database, correlations between quantitative data were determined as in Table 2. Two-sided *p*-values of less than 0.05 were considered statistically significant. All statistical analyses were performed using STATA 12.0 software.

In order to study whether *RND1* expression could be associated with the sensitivity of cancer cells to anticancer therapies (either cytotoxic or targeted agents), we analyzed the potential correlation between *RND1* expression and the cellular response to 20 anticancer agents with the CCLE database (https://portals.broadinstitute.org/ccle). Among the 23 analyzed tumor localizations, the hematological and lymphoid tissue is the tumor type for which the sensitivity to anticancer agents is the most dependent on the *RND1* mRNA levels (Table 2). Figure 7 shows that in these hematological and lymphoid tumors, the lower *RND1* is expressed, the more sensitive the tumor is to inhibitors of growth factor receptors i.e., IGFR1 (AEW541), EGFR (erlotinib), c-met (PF2341066), multiple receptor tyrosine kinase (TKI258), the MAPK pathway (AZD6244, PD-0325901, RAF265), tyrosine kinases i.e., Abl and Alk (nilotinib, TAE684) and γ secretase (L-685458). To determine whether this correlation is specific to RND1 or could be generalized to RND3, we also analyzed the potential correlation between *RND3* expression and the cellular response to 20 anticancer agents in hematological and lymphoid tumor cells. In these tumor cells, the only significant correlation observed between RND3 expression and anticancer agent susceptibility is for PD-0332991, a cdk 4/6 inhibitor (Table 3), showing that the response profile to these agents is specific to a particular RND member. With regards to other tumor localizations, it should be noted that the inverse correlation between sensitivity to a γ secretase inhibitor and *RND1* levels identified in the hematological and lymphoid tumor cells, is also observed in large intestine, ovary, and skin tumor cells (Table 2). Interestingly, the inverse correlation between the sensitivity to the EGFR inhibitor erlotinib and the amount of *RND1* transcripts is also found in large intestine tumor cells. In addition, the more *RND1* is expressed, the more sensitive kidney and lung tumor cells are to the EGFR inhibitor lapatinib. When EGFR is activated, it activates the MAPK pathway. The sensitivity to MAPK inhibitors and the levels of *RND1* is also found in the lung tumor cells (Table 2). These data suggest a role for *RND1* expression in the sensitivity of tumors to EGFR signaling pathway inhibitors. Moreover, according to tumor cell type, positive and negative correlation between the levels of RND1, and the sensitivity to EGFR inhibitors can be found. This necessitates the molecular mechanisms linking RND1 to cancer cell sensitivity.

## 10. Conclusions

In several tumors (triple negative breast cancer, hepatocellular carcinoma, and glioblastoma), RND1 presents the features of a tumor suppressor gene i.e.,: (i) in breast tissue, its loss induces mammary tumorigenesis [10], (ii) its re-expression in tumor cells causes the cell to lose two cancerous properties (invasion/metastasis, proliferation) [10,45,46,47], (iii) its expression is down-regulated in tumors and is inversely correlated with the stage of the tumors [11,45]; and (iv) a weak expression of *RND1* is correlated with a bad prognosis for the patients [10,45,46,47]. However, data from esophageal squamous cell carcinoma refute the idea that *RND1* would be a universal tumor suppressor gene [50]. Apart from its role in oncogenesis, RND1 is a gene induced by anticancer agents and is involved at least in the cellular resistance to cisplatin [45], to sorafenib [46] and camptothecin [53]. The in silico data of sensitivity to anticancer agents, which must be verified with patient samples, offers new research perspectives on RND1 in response to anticancer agents.

In clinical practice, the RND1 rate may be useful to predict patient response to standard therapy. This has already been strongly suggested for breast tumors, hepatocarcinoma, and glioblastoma [10,45,46,47], but could be studied and extended to other tumors. In addition, for patients who do not respond to standard therapy, the RND1 rate in tumor biopsies could be helpful in selecting the most appropriate targeted therapy for each patient. In particular, for patients with hematopoietic and lymphoid tumors, determining the levels of RND1 in tumor tissues would help to evaluate their response to inhibitors of growth factor receptors (IGFR1, EGFR, c-met and multiple receptor tyrosine kinase), MAPK pathway, tyrosine kinases (Abl and Alk), and γ secretase.

## Figures and Tables

**Table 1:** Mutations in the *RND1* gene.

Mutation Type	Cancer Type	Mutation Type	Cancer Type
**Missense**		**Missense**	
R5G	Renal Clear Cell C	M137I	Cutaneous Melanoma
V11A	Stomach A. Diffuse Type Stomach AdenoC	Q142H	Adrenocortical C
V11M	Prostate Neuroendocrine C. Prostate AdenoC	E150D	High-Grade Serous Ovarian Cancer. Serous Ovarian Cancer
C14F	Renal Clear Cell C with sarcomatoid features. High-Grade Serous Ovarian C. Serous Ovarian C	A154T	Uterine Endometrioid C
C14S	Bladder Urothelial C	A156S	Uterine Endometrioid C
V17I	Bladder Urothelial C	G160S	Cutaneous Melanoma
G20V	Cutaneous Melanoma	E162D	Intestinal Type Stomach AdenoC
T45I	Hepatocellular C. Cutaneous Melanoma	L165P	Colorectal AdenoC
V46M	Colorectal AdenoC. Uterine Endometrioid C	E166K	Cutaneous melanoma
E48K	Cutaneous Melanoma	G167D	Astrocytoma
E57G	Acute Myeloid Leukemia	A169V	Cutaneous melanoma
E57K	Lung AdenoC	F180C	Breast C
E58K	Bladder Urothelial C	R181W	Cutaneous melanoma
E62V	Lung Squamous Cell C	S184F	Melanoma
G70R	Breast C	S184T	Renal Clear Cell C
P72S	Cutaneous Squamous Cell C	S184Y	Uterine Endometrioid C
D75H	Breast Invasive Ductal C	M185V	Breast C
D75N	Colorectal AdenoC. Cutaneous Melanoma	P191S	Colorectal AdenoC
S85L	Uterine Endometrioid C	Q196H	Hepatocellular C
D86N	Cutaneous Melanoma	L203F	Lung Squamous Cell C
A87V	Uterine Endometrioid C	R213H	Uterine Carcinosarcoma. Colorectal AdenoC
R96H	Prostate Neuroendocrine C. Uterine Endometrioid C. Colon AdenoC	S214F	Uterine Endometrioid C
E98D	Breast C	I217M	Invasive Breast C. Breast Mixed Ductal and Lobular C. Breast Invasive Ductal C
D101N	Bladder Urothelial C	**Truncating**	
A103T	Stomach AdenoC. Uterine Endometrioid C	A6Pfs * 28	Mucinous AdenoC of the colon and rectum
K106Q	Uterine Endometrioid C	Q8Pfs * 8	Renal Clear Cell C. Stomach AdenoC
W107G	Cutaneous Melanoma	X70_splice	Pancreatic AdenoC
R108K	Cutaneous Melanoma	X107_splice	Esophagogastric AdenoC
R108M	Cutaneous Melanoma	E162 *	Uterine Endometrioid C
E110K	Cutaneous Melanoma	Y164 *	Lung AdenoC
P116S	Bladder Urothelial C	S168 *	Bladder Urothelial C
S117N	Glioblastoma Multiforme	R201 *	Colon AdenoC
T118A	Colorectal AdenoC	R206 *	Uterine Serous/Papillary Serous C
I123F	Pancreatic AdenoC		
K126N	Cervical Squamous Cell C	**Fusion protein**	
R130Q	Uterine Endometrioid C. Colorectal AdenoC. Mucinous AdenoC of the colon and rectum	MLL2-RND1	Esophageal AdenoC
D132G	Mucinous AdenoC of the colon and rectum	DDX23-RND1	Hepatocellular C

cBioportal-generated data depicting the genetic alterations found in human tumors for the *RND1* gene. “C” is the abbreviation for carcinoma. “*” indicates the insertion of a stop codon. The bold format indicates the type of mutations.

**Table 2:** Correlations between *RND1* expression and sensitivity to anticancer agents in several tumor cells.

Anticancer Agents	Drug Target	Auton Ganglia	Bone	Breast	Endom	Hematopoietic	Kidney	Large Intestine	Liver	Lung	Ovary	Pancreas	Pleura	Skin	Aero Digestive
**17AAG**	Hsp90	ns	ns	ns	ns	ns	ns	ns	ns	ns	ns	ns	ns	ns	ns
**AEW541**	IGFR1	ns	0.6273, *p* = 0.039	ns	ns	−0.3068, *p* = 0.009	ns	ns	ns	0.2936, *p* = 0.005	ns	ns	ns	ns	ns
**AZD0530**	Src	ns	ns	ns	ns	ns	ns	ns	ns	ns	ns	ns	−0.8214, *p* = 0.023	ns	ns
**AZD6244**	MEK	ns	ns	ns	ns	−0.4611, *p* = 10^−4^	ns	ns	ns	ns	ns	ns	ns	ns	ns
**Erlotinib**	EGFR	ns	ns	ns	ns	−0.3332, *p* = 0.005	ns	−0.4383, *p* = 0.036	ns	ns	ns	ns	ns	ns	ns
**Irinotecan**	Topo I	ns	ns	ns	ns	ns	ns	ns	ns	ns	ns	ns	ns	ns	ns
**L-685458**	γ secretase	ns	ns	ns	ns	−0.3147, *p* = 0.008	ns	−0.4230, *p* = 0.049	ns	ns	−0.4225, *p* = 0.025	ns	ns	−0.3957, *p* = 0.013	ns
**LBW242**	IAP	ns	ns	ns	ns	ns	ns	ns	ns	ns	ns	ns	ns	ns	ns
**Lapatinib**	EGFR	ns	ns	ns	ns	ns	0.800, *p* = 0.010	ns	ns	0.2649, *p* = 0.011	ns	ns	ns	ns	ns
**Nilotinib**	Abl	ns	0.7, *p* = 0.036	ns	ns	−0.2816, *p* = 0.019	ns	ns	0.7088, *p* = 0.007	ns	ns	ns	ns	ns	ns
**Nutlin-3**	p53-mdm2	ns	ns	ns	ns	ns	ns	ns	ns	ns	ns	ns	ns	ns	ns
**PD-0325901**	MEK	ns	ns	ns	ns	−0.4359, *p* = 0.0001	ns	ns	ns	0.2592, *p* = 0.013	ns	ns	ns	ns	ns
**PD-0332991**	cdk 4/6	−0.8214, *p* = 0.023	ns	ns	0.6571, *p* = 0.008	ns	ns	ns	ns	ns	ns	ns	ns	ns	ns
**PF2341066**	c-met	ns	ns	ns	ns	−0.2580, *p* = 0.030	ns	ns	ns	ns	ns	ns	ns	ns	ns
**PHA-665752**	c-met	ns	ns	ns	ns	ns	ns	ns	ns	ns	ns	ns	ns	ns	ns
**PLX4720**	B-Raf^V600E^	ns	ns	ns	ns	ns	ns	ns	ns	ns	ns	−0.4286, *p* = 0.026	ns	ns	ns
**Paclitaxel**	Microtubules	ns	ns	ns	ns	ns	ns	ns	ns	ns	ns	ns	ns	ns	0.9286, *p* = 0.003
**Panobinostat**	HDAC	ns	ns	ns	ns	ns	ns	−0.4200, *p* = 0.046	ns	ns	ns	ns	0.9286, *p* = 0.003	ns	ns
**RAF265**	B-Raf	ns	ns	ns	ns	−0.3737, *p* = 0.002	ns	ns	ns	ns	ns	ns	ns	ns	ns
**Sorafenib**	Tyrosine kinase	ns	ns	ns	ns	ns	ns	ns	ns	ns	ns	ns	ns	ns	ns
**TAE684**	Alk	ns	ns	−0.4084, *p* = 0.028	ns	−0.2847, *p* = 0.016	ns	ns	ns	ns	ns	ns	ns	ns	ns
**TKI258**	Receptor tyrosine kinase	ns	ns	ns	ns	−0.2764, *p* = 0.020	−0.8333, *p* = 0.005	ns	ns	ns	ns	−0.3996, *p* = 0.035	ns	ns	ns
**Topotecan**	Topo I	ns	ns	ns	ns	ns	ns	ns	ns	ns	ns	ns	ns	ns	ns
**ZD-6474**	VEGFR	ns	ns	ns	ns	ns	ns	ns	ns	ns	ns	ns	ns	ns	ns

Using the CCLE database, correlation between quantitative data were determined by Spearman’s rank correlation coefficient (the cellular sensitivity to anticancerous agents was analyzed with the actarea, which represents the activity area of the drug. The higher the actarea figure, the more sensitive the cell is to drugs). Two-sided *p*-values of less than 0.05 were considered statistically significant. All statistical analyses were performed using STATA 12.0 software. Twenty three tumor localizations were studied. Those for which no correlation has been found do not appear in the table: they correspond to the biliary tract, the central nervous system, the esophagus, the prostate, the salivary gland, the soft tissue, the stomach thyroid, and the urinary tract. Auton: autonomic, endom: endometrium, hematopoietic: hematopoietic and lymphoid tissue, aerodigestive: upper aerodigestive tract. “TopoI” is the abbreviation for Topoisomerase I. ns=not significant. The first value is the correlation coefficient; the second one is the Spearman’s *p*-value.

**Table 3:** Correlations between *RND3* expression and sensitivity to anticancerous agents in hematological and lymphoid tumor cells.

Anticancerous Agents	Correlation Coefficient	*p* Value
17-AAG	0.1237	0.3040
AEW541	0.1882	0.1160
AZD0530	0.0841	0.4855
AZD6244	0.0554	0.6464
Erlotinib	−0.1070	0.3743
Irinotecan	0.0493	0.7311
L-685458	−0.0054	0.9648
LBW242	0.0479	0.6917
Lapatinib	0.1342	0.2646
Nilotinib	0.1233	0.3130
Nutlin-3	0.0628	0.6026
PD-0325901	0.0478	0.6923
PD-0332991	0.2655 *	0.0275
PF2341066	0.1118	0.3532
PHA-665752	0.1113	0.3553
PLX4720	0.0846	0.4831
Paclitaxel	0.0959	0.4262
Panobinostat	−0.0325	0.7879
RAF265	−0.0446	0.7159
Sorafenib	0.0902	0.4544
TAE684	0.1848	0.1229
TKI258	−0.0148	0.9026
Topotecan	0.0772	0.5224
ZD-6474	0.0897	0.4571

Correlation between quantitative data was assessed as in Table 2. Two-sided *p*-values of less than 0.05 were considered statistically significant. All statistical analyses were performed using STATA 12.0 software. *: significant.

## Abbreviations

EMT	Epithelial to mesenchymal transition
FGF	Fibroblast growth factor
FRS2β	FGFR substrate 2 β
FLRT3	Fibronectin Leucine Rich Transmembrane 3
GAP	GTPase-activating protein
GDI	GDP-dissociation inhibitors
GEF	Guanine nucleotide Exchange Factor
Grb7	Growth factor receptor-bound protein 7
lncRNA	Long non-coding RNA
MO	Morpholino antisense oligonucleotide
NFATc1	Nuclear Factor of Activated T Cells
NRP1	Neuropilin 1
PARP-1	Poly (ADP-ribose) polymerase
SCG10	Superior Cervical Ganglion 10
Sema	Semaphorin
STI1	Stress-Inducible Protein
TGF-β	Transforming growth factor beta
VEGF	Vascular endothelial growth factor
